# Development and Validation of a GMP-Compliant High-Pressure Liquid Chromatography Method for the Determination of the Chemical and Radiochemical Purity of [^18^F]PSMA-1007, a PET Tracer for the Imaging of Prostate Cancer

**DOI:** 10.3390/ph14030188

**Published:** 2021-02-25

**Authors:** Ines Katzschmann, Heike Marx, Klaus Kopka, Ute Hennrich

**Affiliations:** 1 German Cancer Research Center (DKFZ), Division of Radiology, Im Neuenheimer Feld 280, 69120 Heidelberg, Germany; i.katzschmann@dkfz.de (I.K.); h.marx@dkfz.de (H.M.); 2 German Cancer Consortium (DKTK), 69120 Heidelberg, Germany; k.kopka@hzdr.de; 3 Helmholz-Zentrum Dresden-Rossendorf (HZDR), Institute of Radiopharmaceutical Cancer Research, Bautzner Landstraße 400, 01328 Dresden, Germany; 4 Faculty of Chemistry and Food Chemistry, Technische Universität Dresden, Mommsenstraße 4, 01062 Dresden, Germany

**Keywords:** [^18^F]PSMA-1007, high performance liquid chromatography (HPLC), validation, GMP, PSMA, prostate cancer, positron emission tomography (PET)

## Abstract

For the PET imaging of prostate cancer, radiotracers targeting the prostate-specific membrane antigen (PSMA) are nowadays used in clinical practice. [^18^F]PSMA-1007, a radiopharmaceutical labeled with fluorine-18, has excellent properties for the detection of prostate cancer. Essential for the human use of a radiotracer is its production and quality control under GMP-compliance. For this purpose, all analytical methods have to be validated. [^18^F]PSMA-1007 is easily radiosynthesized in a one-step procedure and isolated using solid phase extraction (SPE) cartridges followed by formulation of a buffered injection solution and for the determination of its chemical and radiochemical purity a robust, fast and reliable quality control method using radio-HPLC is necessary. After development and optimizations overcoming problems in reproducibility, the here described radio-HPLC method fulfills all acceptance criteria—for e.g., specificity, linearity, and accuracy—and is therefore well suited for the routine quality control of [^18^F]PSMA-1007 before release of the radiopharmaceutical. Recently a European Pharmacopeia monograph for [^18^F]PSMA-1007 was published suggesting a different radio-HPLC method for the determination of its chemical and radiochemical purity. Since the here described method has certain advantages, not least of all easier technical implementation, it can be an attractive alternative to the monograph method. The here described method was successfully validated on several radio-HPLC systems in our lab and used for the analysis of more than 60 batches of [^18^F]PSMA-1007. Using this method, the chemical and radiochemical purity of [^18^F]PSMA-1007 can routinely be evaluated assuring patient safety.

## 1. Introduction

For the PET imaging of prostate cancer, radiotracers targeting the prostate-specific membrane antigen (PSMA) are nowadays used in clinical practice. Amongst the first tracers for this purpose was [^68^Ga]Ga-PSMA-11 which is still the most widely used PET tracer for PSMA imaging [1,2,3,4]. This tracer has a relatively short physical half-life (T_1/2_(^68^Ga) = 68 min) which makes a satellite distribution not feasible: the tracer has to be prepared on site or at least nearby. A huge advantage of using ^68^Ga as PET radionuclide is its availability from a generator which enables also small production sites without a cyclotron to produce their own PET tracers. However, due to the capacity of the ^68^Ge/^68^Ga-generator used for the production of a ^68^Ga-labelled radiotracer, the amount of radioactivity which can be produced in one batch is limited in comparison to fluorine-18 labelled tracers. This in turn is limiting the number of patients which can be imaged with one batch necessitating a new tracer production when aiming to image more than 2–3 patients a day. Even employing a large-scale production of ^68^Ga with a cyclotron, which is currently under investigation [5], a satellite distribution of the tracer is doubtful. Using a PET tracer labeled with fluorine-18 these disadvantages can be overcome. With a half-life of 110 min for fluorine-18, a distribution of the radiotracer in a satellite concept (centralized production and distribution to other sites) is possible. Moreover, a large scale production with resulting high amounts of radioactivity enable the imaging of multiple patients with one batch of radiotracer. One such tracer for the imaging of PSMA is [^18^F]PSMA-1007, a radiopharmaceutical labeled with fluorine-18, which has excellent properties for the detection of prostate cancer [6,7]. During the last years multiple studies have been performed validating its suitability and in some cases superiority over other PSMA-tracers [8,9,10,11,12].

In order to use a radiotracer in human applications, this tracer has to be produced and its quality ascertained under full GMP-compliance. The production should take place fully automated in a cleanroom environment. The fully automated production of [^18^F]PSMA-1007 was established on several commercially available synthesizers [13,14,15]. Before use in human application each batch of the produced radiopharmaceutical has to be evaluated in regard to its quality before release. For some commonly used radiopharmaceuticals, recently also [^18^F]PSMA-1007, parameters to be evaluated as well as their acceptance criteria and suitable analytical methods are described in monographs of the current European Pharmacopeia [16]. Typical parameters to be analyzed for a radiopharmaceutical are the chemical and radiochemical purity, the pH value, the radionuclidic purity, bacterial endotoxins, integrity of the sterile filter, and sterility of the injection solution. Due to the limited half-life of the radiotracer some of these tests, e.g., sterility, may be evaluated after release. The methods for the analysis of parameters before release have to be fast and reliable in order to complete quality control in ideally less than 30 min. Concerning the chemical purity of a fluorine-18 labeled radiotracer several methods should be used: HPLC (high performance liquid chromatography) for determination of related substances, a spot test for content determination of the phase transfer catalyst used in the radiolabeling reaction, and gas chromatography for determination of residual solvents. The radiochemical purity should be accessed using radio-HPLC and radio-TLC (thin layer chromatography). All methods used for the quality control have to be validated assuring full GMP-compliance. In this work a fast and reliable radio-HPLC method for the determination of the chemical and radiochemical purity of [^18^F]PSMA-1007 injection solution was developed and validated on several HPLC systems in our lab. This method has certain advantages over the recently published monograph method and can be an attractive alternative. The successfully validated radio-HPLC method was routinely used for the analysis and subsequent release of more than 60 batches of [^18^F]PSMA-1007 over more than one year assuring patient safety.

## 2. Results and Discussion

### 2.1. Recommended Acceptance Criteria for the Chemical and Radiochemical Purity of [^18^F]PSMA-1007 Determined by Radio-HPLC

For the determination of the chemical and radiochemical purity of a radiopharmaceutical, acceptance criteria using different analytical methods have to be defined. Since typical radionuclides used for the labeling of radiopharmaceuticals are no-carrier-added (n.c.a.), the product solution will contain varying amounts of the non-radioactively labeled compound: in case of [^18^F]PSMA-1007 this is the reference compound PSMA-1007. Additionally, other impurities may be formed during the labelling reaction which may be known or unknown impurities. The chemical purity of [^18^F]PSMA-1007 can be accessed by HPLC using UV detection. The radiochemical purity can be evaluated using the same analytical method by having a radioactivity detector in series. Typical methods for the quality control of radiopharmaceuticals include radio-HPLC, radio-TLC, and gas chromatography for determination of residual solvents. For some commonly used radiopharmaceuticals—e.g., [^18^F]FDG, [^18^F]FLT, and [^18^F]FET—these acceptance criteria as well as suitable analytical methods are described in monographs of the current European Pharmacopeia [16]. For [^18^F]PSMA-1007 a monograph (“PSMA-1007 (18F) injection”; 07/2021:3116) detailing its acceptance criteria has recently been published [17] but has not been available during the development and validation of the here described radio-HPLC method. Table 1 summarizes recommended acceptance criteria for the chemical and radiochemical purity of [^18^F]PSMA-1007 established in our lab as already published [13] which have now been confirmed by the release of monograph 3116. Only for the radiochemical purity our acceptance limit is higher (≥95% vs. ≥91%).

The limit value for the unlabeled compound PSMA-1007 (reference standard) in the [^18^F]PSMA-1007 injection solution is recommended as 10 µg/mL (0.1 mg/V_max_) which has recently also been defined in the monograph of [^18^F]PSMA-1007. The peak area of this reference solution has to be determined in calibration measurements and routinely checked before analyzing the quality control sample of [^18^F]PSMA-1007 injection solution since this area is the basis for the comparison of peak areas of other impurities: the peak area of any impurity has to be less than or equal to the peak area of a reference solution of PSMA-1007 (10 µg/mL). The sum of the peak areas of all impurities including PSMA-1007 has to be less than or equal to 5 × the peak area of the reference solution. Peak areas ≤ 0.3 x the peak area of the reference solution can be disregarded.

The radiochemical purity of [^18^F]PSMA-1007 is recommended to be ≥95%, also in accordance with other established radiopharmaceuticals. Radiochemical impurities have to be ≤5%. Recently a radiochemical purity of ≥91 % for [^18^F]PSMA-1007 has been suggested in the monograph 3116 where the impurity caused by [^18^F]fluoride has to be ≤5%. This is fulfilled by the here recommended acceptance criterion. 

### 2.2. Development and Validation of the Radio-HPLC Method

For the routine use of an analytical method for the quality control of radiopharmaceuticals, this method has to be GMP-compliant. In order to ensure that, after development the method has to be validated regarding different parameters according to the ICH Q2 (R1) guideline “Validation of Analytical Procedures” [18]. For the analysis of radiopharmaceuticals not only the chemical purity has to be taken into consideration but also the radiochemical purity of the product solution. This makes them special which may result in some modifications of the ICH guideline concerning radiochemical parameters. To this effect, a guideline for the validation of analytical methods for radiopharmaceuticals was published by the EANM [19]. While developing and validating our radio-HPLC method for [^18^F]PSMA-1007, both guidelines were taken into consideration. Using this analytical method, the following specification parameters of [^18^F]PSMA-1007 injection solution were determined: chemical identity of PSMA-1007, radiochemical identity of [^18^F]PSMA-1007, amount of PSMA-1007 as well as chemical and radiochemical impurities of the injection solution. For GMP-compliance all these parameters have to be validated. The here successfully validated radio-HPLC method for the determination of the chemical and radiochemical purity of [^18^F]PSMA-1007 has the following parameters (Table 2):

This method is different to the recently suggested one in the Eur. Ph. monograph “PSMA-1007 (18F) Injection” [17] whose parameters are listed in Table 3 for comparison.

The most severe difference between the two methods is the mobile phase: while the in this work validated method uses the addition of 0.1% TFA to the aqueous phase, the monograph method uses a phosphate buffer. A salt buffer has the huge disadvantage that it may crystallize in the HPLC system resulting in blockages. To reduce this risk the system has to be cleaned very thoroughly after the analysis which takes some time. Moreover, the preparation of a salt buffer is more elaborate, error-prone and time consuming in comparison to the addition of TFA to water. Another difference is the run time of the method which has to be at least double the retention time of the analyte. In the here validated method it takes 19 min to again reach the starting conditions of the method in comparison to approximately 25 min for the monograph method. Since the HPLC run has to be completed before release of the radiopharmaceutical the shorter the run time the better. Still another difference between the two methods is the UV wavelength chosen for the measurements (254 nm vs. 225 nm): generally, the adsorption of solvents is higher at lower wavelengths which may result in an increase of baseline noise. A parameter which makes the monograph method technically more difficult to implement, is the slightly elevated temperature of 30 °C versus room temperature in the here presented method. This necessitates a column oven which may not be present in every HPLC system representing a knock out criterium for these systems. Regarding the column stationary phase, the monolithic as well as the core-shell phase technology show high resolution, productivity and sensitivity. Comparing both methods regarding their differences, the here developed and validated method has certain advantages over the monograph method which may make it attractive to users worldwide. European users which have to consider the monograph for [^18^F]PSMA-1007 may use this method, too, when explaining its advantages to their authorities and showing that it is suitable.

The validation of the here developed method along with optimizations to overcome problems in reproducibility, which have also been encountered by other groups, will be described for two different HPLC systems, an UltiMate 3000 system (Thermo Fisher Scientific GmbH, Dreieich, Germany) as well as an ICS5000 system (Thermo Fisher Scientific GmbH, Dreieich, Germany). Both of them have subsequently been used routinely for the quality control of [^18^F]PSMA-1007 in our lab. The following parameters, whose acceptance criteria are based on monographs for radiopharmaceuticals as well as guidelines for method validation [16,17,18,19], have been analyzed during the validation (Table 4):

After successful validation, the results for chemical and radiochemical purity on both systems have been compared for several batches of [^18^F]PSMA-1007.

### 2.3. Development and Validation of the HPLC Method Concerning Chemical Purity

#### 2.3.1. Chemical Identity of PSMA-1007, Possible Impurities and Specificity

**Figure 1:**
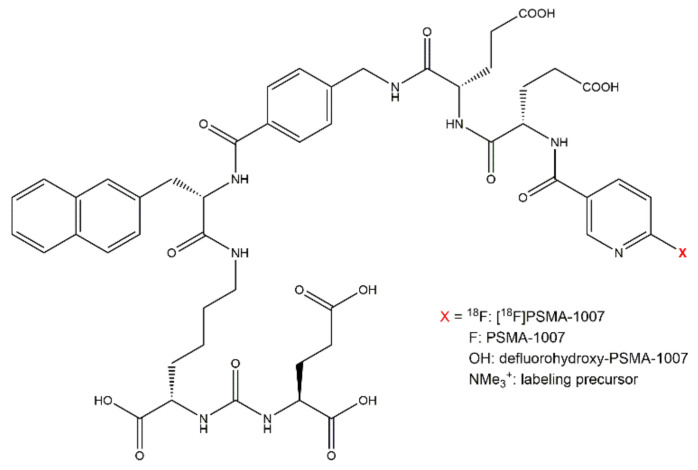
Molecular structure of [^18^F]PSMA-1007, PSMA-1007 as well as known impurities.

For the evaluation of the chemical purity of the [^18^F]PSMA-1007 injection solution the chemical identity of PSMA-1007 as well as known impurities have to be validated. Furthermore, unknown impurities may occur in the product solution. With an external reference standard, the chemical identity of PSMA-1007 can be verified using its retention time. The retention time of the product should be at least 3 times the systems dead volume. For the validation of the suitability of the method to determine the chemical identity of PSMA-1007, a reference standard solution of PSMA-1007 was injected. Possible known impurities which may occur in the injection solution of [^18^F]PSMA-1007 can be the labeling precursor of PSMA-1007 (trimethyl-ammonium-precursor for direct labeling) and defluorohydroxy-PSMA-1007. These compounds were also separately injected and identified by means of their retention times. Figure 1 shows the molecular structures of [^18^F]PSMA-1007, PSMA-1007 as well as possible known impurities.

Using the UltiMate 3000 system, the retention times were 7.77 min for PSMA-1007, 6.32 min for the labeling precursor, and 6.17 min for defluorohydroxy-PSMA-1007. In comparison to the systems dead volume of 0.6 min, all compounds fulfill the criterion that their retention time has to be at least 3 times the dead volume. Using the ICS5000 system, the retention times were 8.35 min for PSMA-1007 and 6.78 min for the labeling precursor. Also in this case the criterion of at least 3 times the dead volume (0.7 min) was fulfilled. Due to its non-availability at that time, the identity of defluorohydroxy-PSMA-1007 was not evaluated on this system. Since the other retention times between the two systems were comparable, it can be assumed that defluorohydroxy-PSMA-1007 would have a similar retention time as the labeling precursor on the ICS5000 system. On both systems the chemical identities of PSMA-1007 and known impurities were identified by means of their retention times. 

Furthermore unknown impurities may occur in the [^18^F]PSMA-1007 injection solution. Since their identity is not known, they cannot be injected separately. As recommended, impurities may be disregarded if they are below the limit of 0.3 × the peak area of the reference solution of PSMA-1007 (10 µg/mL). Nonetheless unknown as well as known impurities have to be adequately separated from PSMA-1007 in order to not interfere with the determination of its amount.

The specificity which ensures that unknown as well as known impurities are suitably separated from PSMA-1007, can be validated by injection of a solution containing all known impurities and determination of the resolution between the compounds. In reference to the Eur. Ph. monograph of [^18^F]FET [20], the resolution between PSMA-1007 and related substances has to be at least 2.0 for both compounds to be suitably separated. For the UltiMate 3000 system the resolution between PSMA-1007 and the labeling precursor was 13.6 while it was 15.1 between PSMA-1007 and defluorohydroxy-PSMA-1007. For the ICS5000 system the resolution to the labeling precursor was 13.3. The specificity of the method for known impurities was thus fulfilled on both systems. A baseline separation between the two known impurities, the labeling precursor and defluorohydroxy-PSMA-1007, which is recommended in the recently released monograph of [^18^F]PSMA-1007 [17], is not given using this HPLC method. Since the exact amount of these impurities does not have to be determined but only generally the peak area of impurities (≤peak area of reference standard peak), this is of no further concern. The potential addition of both peak areas as one impurity would increase this peak area. This in turn makes a possible violation of the limit more probable not less therefore securing the safety of the product. In order to validate a suitable resolution of PSMA-1007 and unknown impurities, a typical batch of the injection solution of [^18^F]PSMA-1007 was analyzed. The UV-chromatogram showed a resolution of 4.0 and 3.8 to the neighboring peaks of PSMA-1007 on the UltiMate 3000 system and a resolution of 3.9 and 5.2 on the ICS5000 system. The acceptance criterion of at least 2.0 for the resolution to unknown impurities was therefore also fulfilled on both systems. The specificity of the method for known as well as unknown impurities to PSMA-1007 was proven.

#### 2.3.2. Precision and Repeatability

A very important parameter for the suitability of a method for routine measurements is its precision or repeatability. The precision expresses the closeness of the results when repeatedly measuring a homogenous sample with the same analytical method. For validating the precision of the method, the samples used for the determination of the linearity were injected 4–5 times for each concentration and the relative standard deviations (RSD) of the peak areas of each level were calculated which should be less than 2%.

When first starting the [^18^F]PSMA-1007 production, the injection solution contained 90% physiological saline (0.9% NaCl) and 10% ethanol as well as sodium ascorbate for stabilization. For the validation the reference standard PSMA-1007 was solved in physiological saline/ethanol (90:10 *v*/*v*) without sodium ascorbate because its peak eluted at 0.6 min (dead volume) and was huge in comparison to the PSMA-1007 peak. Measurements of PSMA-1007 with or without sodium ascorbate were otherwise completely comparable. Since solubility of PSMA-1007 in aqueous solution is poor, the reference standard was first solved in 50 µL DMSO and then diluted accordingly. Using normal plastic tubes and pipette tips for dilution resulted in completely unreproducible results which could be expected since droplet formation in the tips was seen during dilution. This problem was solved by using so called “protein low bind” tubes and tips for preparation of the standard solutions.

The first system used for validation of the HPLC method for [^18^F]PSMA-1007 in our lab was the UltiMate 3000 system. Table 5 summarizes the results of the measurements of 5 different concentration levels of PSMA-1007. The precision of the method was very good for all concentration levels. Using the ICS5000 system, the RSDs of peak areas of PSMA-1007 were also well below the acceptance criterion of 2%.

But when using this system for the analysis of [^18^F]PSMA-1007 batches and comparing them to the results of the same batches on the UltiMate 3000 system huge discrepancies occurred. Peak areas during validation measurements and measurements of the reference standard solution were approximately half the size for the ICS5000 system used compared to those of the UltiMate 3000 system. This was not the case for measurements of [^18^F]PSMA-1007 batches which were almost comparable in size. Therefore, when measuring a reference standard solution of PSMA-1007 before the batch analysis on both systems, the results were comparable (e.g., 10.38 µg/mL (ICS5000) vs. 10.24 µg/mL (UltiMate 3000)). However, the following batch analysis resulted in apparently much higher amounts of PSMA-1007 when measuring with the ICS5000 (e.g., 17.44 µg/mL vs. 8.79 µg/mL) leading to a very poor intermediate precision between the two systems. Normally an ICS5000 system is used for ion chromatography and also other detectors, e.g., electrochemical detectors, are used. Since production of [^18^F]FDG was cancelled in our lab, we reused the ICS5000 as a HPLC system by bypassing the electrochemical detector and using only the UV and radioactivity detectors. A huge difference between the ICS5000 and the UltiMate 3000 system is the mode of injection by the respective autosampler: for the injection of a sample in the ICS5000 system the sample is first drawn into a buffer line and then pushed through the sample loop onto the column (push mode) [21]. In the UltiMate 3000 system a split-loop injection principle is used: the sample is drawn directly into the sample loop and then injected onto the column, the syringe and sample loop are part of the chromatographic system [22]. This different kind of injection types presumably lead to the discrepancies observed. During the validation measurements using the reference standard solutions in physiological saline without the addition of sodium ascorbate, part of the standard remained in the buffer line probably due to slight insolubilities resulting in smaller peak areas and a wrong calibration curve. When injecting the [^18^F]PSMA-1007 batches (physiological saline containing sodium ascorbate), no sample remained in the buffer line leading to higher peak areas and seemingly higher amounts of PSMA-1007. Due to direct injection on the UltiMate 3000 system such effects were not observed, leading to comparable peak areas between reference standard samples and [^18^F]PSMA-1007 samples. 

During the investigation of the discrepancy between the two systems and trying to find a solution for it, the injection solution matrix of [^18^F]PSMA-1007 was changed from 90% physiological saline (0.9% NaCl) and 10% ethanol as well as sodium ascorbate for stabilization to 90% PBS (phosphate buffered saline) and 10% ethanol necessitating a new validation of the method concerning certain parameters using reference standard solutions in the new matrix. Again, five different concentration levels of PSMA-1007 were analyzed and the precision calculated as the RSD between the determined peak areas (Table 6). For both systems the same samples were used. The precision on the UltiMate 3000 was very good. Even though the RSDs for the ISS5000 system are objectively higher than the ones obtained from the measurements of samples in physiological saline, with this method no discrepancies between reference standard measurements and batch measurements could be observed and also intermediate precision of the two systems was very good (see Section 2.5. Comparison of batch results). The presence of phosphate salts in the injection solution apparently prevented slight insolubilities of PSMA-1007 in the buffer line of the ICS5000 autosampler. The acceptance criterion of RSDs < 2% was fulfilled for both systems. 

#### 2.3.3. Range and Linearity

As described in Section 2.2., the suggested limit for the amount of PSMA-1007 in the injection solution is 100 µg per day which has been confirmed by the recently published monograph of [^18^F]PSMA-1007 [17]. At a maximal injection volume of 10 mL per patient this results in a limit value of 10 µg/mL. This value is the basis for the determination of the PSMA-1007 content in the validation of the method. The range of an analytical method should cover 80–120 % of the limit value [18] which for PSMA-1007 results in a range of 8–12 µg/mL. Over this range the method should have an acceptable measure of linearity, accuracy and precision. For validation so that the lower limit of the range is not below the limit of quantitation (LOQ), in a chromatogram of the lowest concentration level the signal-to-noise-ratio (S/N) of PSMA-1007 has to be higher than 10. 

**Figure 2:**
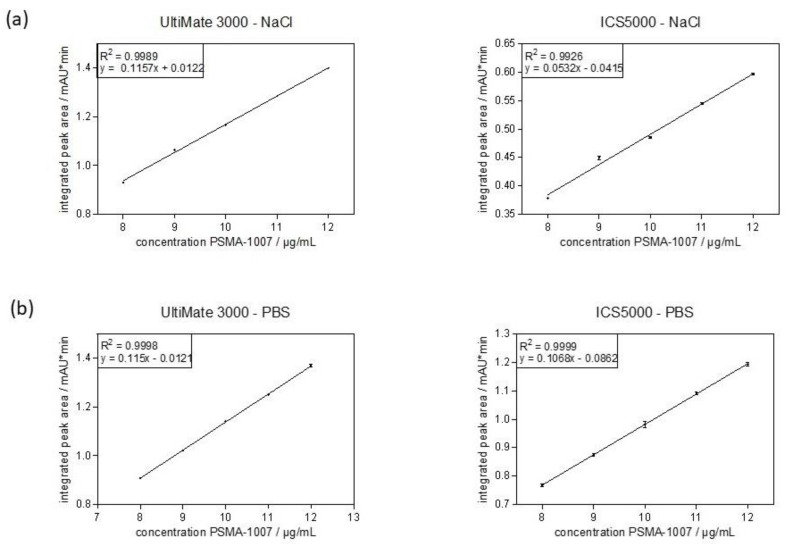
Calibration curve obtained from average integrated peak areas (mean ± standard deviation) of different concentrations of PSMA-1007 over the range of the method. (**a**) reference standard solutions in physiological saline. (**b**) reference standard solutions in PBS.

In order to quantitatively determine the amount of PSMA-1007 in the injection solution, the linearity of the method has to be validated. For this, five concentrations of PSMA-1007 reference standard solution were examined and injected four (UltiMate 3000) or five (ICS5000) times each. For the linearity determination of the method, the average values of these peak areas were plotted against the known concentration and a linear regression was performed. Again, the first validation was performed with standard solutions in physiological saline (0.9%) containing 10% ethanol (Figure 2a). The calibration curve for both systems was linear over the range and confirmed statistically. The correlation coefficients (*R*^2^) were 0.9989 (UltiMate 3000) and 0.9968 (ICS5000), fulfilling the acceptance criterion of *R*^2^ > 0.99. The second validation was performed with standard solutions in PBS containing 10% ethanol (Figure 2b). Using these standard solutions, the calibration curves for both systems were excellent with correlation coefficients of 0.9998 (UltiMate 3000) and 0.9999 (ICS5000) also fulfilling the acceptance criterion for linearity of the method. The linearity of the method over the range was successfully validated on both systems.

Analyzing the chromatograms of the lowest concentration level of the range of PSMA-1007 (8 µg/mL) in order to confirm that it is not below the limit of quantitation (LOQ), the signal-to-noise-ratio (S/N) was determined for both systems and both injection matrices. For the standard solution in physiological saline the S/Ns were 239 (UltiMate 3000) and 64 (ICS5000), respectively. For the standard solution in PBS the S/Ns were 565 (UltiMate 3000) and 248 (ICS5000), respectively. Therefore, for both systems and both matrices the acceptance criterion of S/N > 10 was easily fulfilled.

#### 2.3.4. Limit of Quantitation

To determine the limit of quantitation of the analytical method, the standard solutions of PSMA-1007 were further diluted until the LOQ was reached. These measurements were also conducted for both systems and both injection matrices. The S/N ratios were calculated by the Chromeleon 7.2 software. Table 7 summarizes the results.

With the exception of the solutions in physiological saline on the ICS5000 system, the LOQ for PSMA-1007 using the validated method was 0.75 µg/mL which is well below the lowest concentration level of the range (8 µg/mL). Given the problems of the slight insolubility of the PSMA-1007 standards in physiological saline when injecting on the ICS5000 system as discussed above, the lower sensitivity (LOQ 1.5 µg/mL) of this method could be expected. Concerning the disregard limit for impurities (0.3 x area of reference peak PSMA-1007 (10 µg/mL) ≙ 3 µg/mL), it is essential that this peak area is detectable which was indeed feasible. 

#### 2.3.5. Accuracy

The accuracy of a method is a measure for the conformity of a determined value with the assumed or accepted true value. The accuracy can be either surmised from the parameters specificity, linearity and precision when the reference matrix has no influence on the measurement or can be calculated as the average bias (positive or negative deviation from 100%). Table 8 and Table 9 summarize the calculated concentrations of the samples as well as their average bias for all concentrations of PSMA-1007 on both systems and in both injection matrices.

For the reference standards in physiological saline and the UltiMate 3000 system the average bias for the different concentrations were between −0.05% and 0.97% while they were between 0.03% and 2.53% for the ICS5000 system. As can be seen, the accuracy of the method with reference standards in physiological saline was significantly less for the ISC5000 system in comparison to the UltiMate 3000 system. When evaluating the reference standards in PBS solution, the average bias for the UltiMate 3000 system was between −0.03% and 0.27% whereas values between −0.01% and 0.28% were achieved for the ICS5000 system. For both systems the accuracy of the method using reference standard solutions in PBS was excellent, comparable to each other and higher in comparison to the standard solutions in physiological saline. The acceptance criterion of an average bias < 5% was easily fulfilled validating the accuracy of the method.

All parameters for establishing the HPLC method concerning the chemical purity of [^18^F]PSMA-1007 injection solution were successfully validated. While for the UltiMate 3000 system the matrix of the PSMA-1007 standard solutions made no difference, this was not the case for the ICS5000 system. Using this system, it was essential to use the standard solutions in PBS.

### 2.4. Validation of the HPLC Method Concerning Radiochemical Purity

Another very important specification parameter of a radiopharmaceutical is its radiochemical purity which can be determined using radio-HPLC and radio-TLC. For validation of a radio-HPLC method, some modifications of the ICH guideline concerning radiochemical parameters are necessary. To this point, a guideline for the validation of analytical methods for radiopharmaceuticals was published by the EANM [19]. While developing and validating our radio-HPLC method for [^18^F]PSMA-1007, both guidelines were taken into consideration. The following parameters were evaluated: radiochemical identity of [^18^F]PSMA-1007, specificity, recovery, linearity, limit of quantitation, and intermediate precision.

#### 2.4.1. Radiochemical Identity of [^18^F]PSMA-1007, Possible Impurities and Specificity

**Figure 3:**
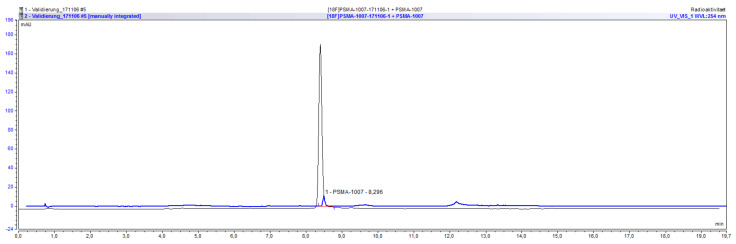
Overlay of UV and radiochromatogram of [^18^F]PSMA-1007 co-injected with PSMA-1007.

The radiochemical identity of a compound can be validated by co-injection of an external standard and the comparison of their retention times. The retention time of the radiolabeled compound can be either larger or smaller than that of the external standard, depending on the arrangement of the UV and radiochemical detectors. For the validation that the method is suitable for determining the radiochemical identity of [^18^F]PSMA-1007, its retention time was compared to that of co-injected reference standard PSMA-1007. For the UltiMate 3000 system the retention time of [^18^F]PSMA-1007 was 7.82 min whereas it was 7.73 min for PSMA-1007. The deviation between the two times is therefore 0.09 min or 1.15% in relation to the retention time of the standard compound. Considering the arrangement of both detectors (1. UV and 2. radio) a shift to higher retention times for the radiochemical compound is to be expected. For the ICS5000 system the retention time of [^18^F]PSMA-1007 was 8.39 min whereas it was 8.30 min for PSMA-1007. The deviation between the two times is therefore 0.09 min or 1.08% in relation to the retention time of the standard compound. Moreover, for this system considering the arrangement of both detectors (1. UV and 2. radio) a shift to higher retention times for the radiochemical compound is to be expected. The acceptance criterion that the deviation between retention times has to be less than 10% is fulfilled for both systems and the method is suitable for the determination of the radiochemical identity of [^18^F]PSMA-1007. An overlay of the respective UV and radiochromatogram of the co-injection of PSMA-1007 reference standard and a batch of [^18^F]PSMA-1007 on the ICS5000 system is shown in Figure 3.

Both the chemical purity and the radiochemical purity for each batch of [^18^F]PSMA-1007 has to be determined. Therefore the method has to be specific for known and unknown radiochemical impurities of [^18^F]PSMA-1007. A known impurity of [^18^F]PSMA-1007 is unreacted [^18^F]fluoride. In order to confirm a suitable resolution between [^18^F]PSMA-1007 and [^18^F]fluoride, a sample of [^18^F]PSMA-1007 injection solution was spiked with [^18^F]fluoride. For the UltiMate 3000 system the retention time for [^18^F]fluoride was 0.65 min resulting in a resolution of 48.8 to [^18^F]PSMA-1007. For the ICS5000 system the retention time for [^18^F]fluoride was 0.66 min resulting in a resolution 49.2 to [^18^F]PSMA-1007. Additionally, to known impurities also unknown impurities may occur in the injection solution. For validating a suitable specificity of the method for unknown impurities, the same batch samples of [^18^F]PSMA-1007 injection solution were analyzed and the resolution to neighboring peaks was determined. In case of the UltiMate 3000 system, the resolution to the neighboring peak was 2.8 and for the ICS5000 system it was 5.47. Therefore the acceptance criterion of a resolution of ≥1.5 was fulfilled for known as well as unknown impurities on both systems, validating a suitable specificity of the method for the determination of the radiochemical purity of [^18^F]PSMA-1007 injection solution.

#### 2.4.2. Recovery

In radiochemical HPLC analysis it is a well-known problem that some radiochemical compounds may be partially retained on the HPLC column therefore falsifying the results of the radiochemical purity of the injection solution. To confirm that this is not the case for a given method, the recovery of a sample can be determined. For this purpose, a known amount of radioactivity is injected, the whole HPLC run is collected and its amount of radioactivity is measured. After half-life correction the percentage of recovered radioactivity can be calculated. The recovery of [^18^F]PSMA-1007 was only tested on the UltiMate 3000 system and was very good with 98.2% fulfilling the acceptance criterion of a recovery of >95%. The deviation from 100% may be due to an error in measurement of the radioactivity which can be expected since the measurement geometry of the sample solution and the recovered HPLC run differs.

#### 2.4.3. Linearity

A very important parameter of an analytical method is the linearity of the used detector over the range of the method. The linearity was not only validated for the UV detector (see Section 2.3.3) but also for the radioactivity detector. Routinely all radioactivity detectors in our lab get tested for their linearity. For this purpose, an equal volume from the same radiopharmaceutical solution is injected four times at specified time intervals. The time intervals depend on the half-life of the radionuclide so that an expected amount of radioactivity remains in the sample and is injected (e.g., 70%, 50% and 33% of radioactivity). The percentage of the integrated peak area is calculated ((area(t_x_)/area(t_0_)) × 100) and compared to the expected value. From these values the linearity of the detector can be determined. For the determination of the linearity of the radiodetector of each HPLC system three independent measurement series were evaluated and the correlation coefficients of the linear regressions were determined. In case of the UltiMate 3000 system R^2^ for the radiodetector was 0.998 and for the ICS5000 system it was 0.9999. The acceptance criterion of *R*^2^ ≥ 0.99 was fulfilled for both systems.

**Figure 4:**
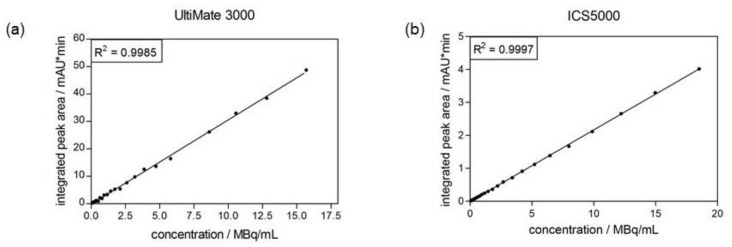
Calibration curve of radiodetector obtained from determination of its limit of quantitation. (**a**) radiodetector of UltiMate 3000 system. (**b**) radiodetector of ICS5000 system.

The linearity of the radiodetector can also be validated during the determination of the limit of quantitation (see Section 2.4.4) of the radiodetector. Linear regression of the obtained integrated peak areas against the calculated radioactive concentration resulted in a correlation coefficient of 0.9985 for the UltiMate 3000 system and of 0.9997 for the ICS5000 system. Both linear regressions are shown in Figure 4.

#### 2.4.4. Limit of Quantitation

As the expected amounts of radioactivity for radiochemical impurities of a radiopharmaceutical are low, another important parameter of the method is the limit of quantitation of the systems radiodetector. This can be evaluated by repeated injection of the same radiopharmaceutical solution at specified time intervals. Due to radioactive decay the amount of radioactivity declines and the sample can be measured until the limit of quantitation (S/N > 10) is reached. The LOQ was determined for both systems and the S/N ratio was calculated by Chromeleon 7.2 software in each radiochromatogram. The LOQ for the UltiMate 3000 system was 6.45 kBq while it was 4.6 kBq for the ICS5000 system. It is desirable to determine the radiochemical purity of a radiopharmaceutical within ±0.5% and this should be achievable also at the lowest radioactivity concentration allowed in the specification of the product. In the case of [^18^F]PSMA-1007 in our lab, the specified radioactivity range is 100–2000 MBq/mL. With the achieved LOQ on both systems, a change in radiochemical purity of ±0.5% should be measurable.

#### 2.4.5. Intermediate Precision

Another parameter to be validated in an analytical method is the intermediate precision which can take the analyst into account: different operators may achieve different results. Since injection onto the HPLC systems is automatic in our lab, differences in injection due to the analyst will not occur. However, in contrast to the automatic integration of the software in the UV chromatograms, the radiochromatograms often need to be re-integrated. To validate the intermediate precision for this re-integration by different analysts, radiochromatograms of 10 batches of [^18^F]PSMA-1007 were independently integrated by three different persons who routinely perform quality control of this radiotracer. The intermediate precision for the determination of the radiochemical purity of [^18^F]PSMA-1007 by three different analysts was very good with RSDs between 0.22% and 0.57% fulfilling the acceptance criterion of < 2%. The method is well suited for use by different operators.

All parameters concerning the radiochemical purity of [^18^F]PSMA-1007 were successfully validated on both systems. The radio-HPLC method for the determination of the chemical and radiochemical purity of [^18^F]PSMA-1007 injection solution was successfully validated, enabling its use for a GMP-compliant routine quality control of the radiopharmaceutical. For the ICS5000 system it was essential that the PSMA-1007 standard solutions were prepared in PBS. Generally, the method using standard solutions in PBS gave better results for both HPLC systems.

### 2.5. Comparison of Batch Results of [^18^F]PSMA-1007

**Figure 5:**
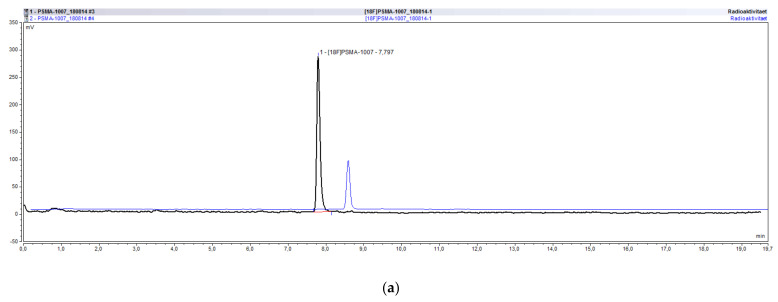

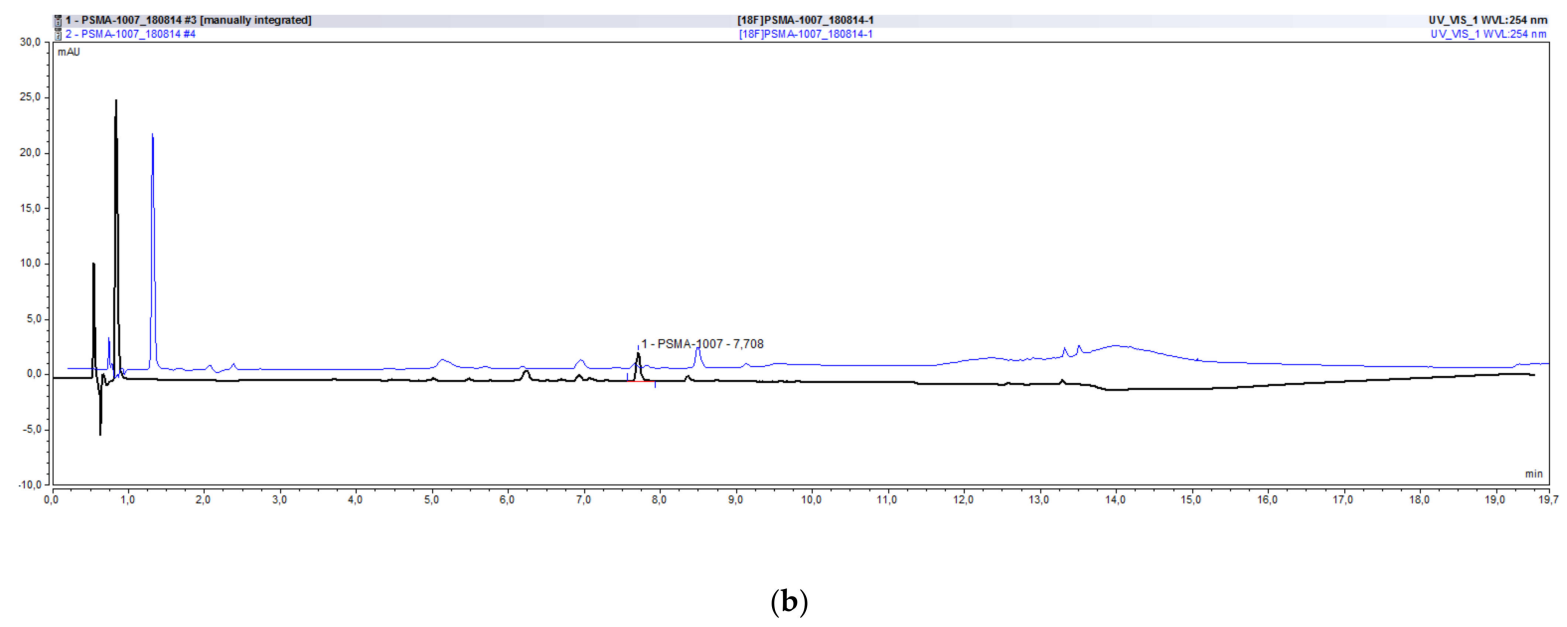
Overlay of typical chromatograms of the same batch of [^18^F]PSMA-1007 analyzed on the UltiMate 3000 and the ICS5000 system. (**a**) radioactivity chromatograms. (**b**) UV chromatograms.

Using the successfully validated radio-HPLC method for the determination of the chemical and radiochemical purity of [^18^F]PSMA-1007, more than 60 batches were analyzed in our lab. Sometimes both systems, the UltiMate 3000 as well as the ICS5000, were used in parallel for the analysis. Figure 5 exemplarily shows an overlay of the radioactivity chromatograms (a) and UV chromatograms (b) of the same batch of [^18^F]PSMA-1007 injection solution analyzed on both systems demonstrating the excellent comparability of both systems.

A comparison of the results for the chemical as well as radiochemical purity for 5 different batches of [^18^F]PSMA-1007 on both systems is summarized in Table 10.

The conformance of the batch results for the chemical as well as the radiochemical purity of [^18^F]PSMA-1007 on both systems is excellent, validating the methods suitability for the routine quality control of this radiotracer.

## 3. Materials and Methods

### 3.1. Reagents and Equipment

The UltiMate 3000 radio-HPLC system from Thermo Fisher Scientific GmbH (Dreieich, Germany) consisted of an LPG-3400SD pump, a WPS-3000SL autosampler, a variable wavelength VWD-3400 UV/VIS detector, an UCI-50 chromatography interface and a Gabi Star radioactivity detector from Elysia-raytest (Straubenhard, Germany). The ICS5000 system from Thermo Fisher Scientific GmbH (Dreieich, Germany) consisted of an ICS5000(+) SD gradient pump, an ICS5000(+) DC detector compartment, an AS-AP autosampler, an VWD UV detector, an UCI-50 chromatography interface and a Gabi Star radioactivity detector from Elysia-raytest (Straubenhard, Germany). Analysis of HPLC data was performed with Chromeleon 7.2.3 software (Thermo Fisher Scientific GmbH, Dreieich, Germany).

PSMA-1007 reference standard, PSMA-1007 labeling precursor, and defluorohydroxy-PSMA-1007 as well as PBS buffer solution and 30% EtOH were obtained from ABX advanced biochemical compounds GmbH (Radeberg, Germany). The exact composition of the PBS buffer solution was not described by ABX, only its constituents (potassium chloride, sodium chloride, potassium dihydrogen phosphate, and anhydrous disodium hydrogen phosphate). Physiological saline solution (0.9%) was obtained from Braun (Melsungen, Germany) and DMSO from Sigma-Aldrich GmbH (Taufkirchen, Germany). All chemicals were used without further purification. [^18^F]PSMA-1007 was synthesized on a Nuclear Interface Tracerlab FX FN system as described earlier [13].

### 3.2. Preparation of Reference Standard Solutions

As solvent for the reference standard solutions either a physiological saline based mixture or a PBS based mixture was applied. These matrices were achieved by mixing of 30% EtOH (8 mL) with either 0.9% NaCl (22 mL) or PBS (22 mL) representing the composition of the [^18^F]PSMA-1007 injection solution. Reference standard PSMA-1007 (1 mg) was dissolved in 50 µL DMSO and diluted with one of the matrices to achieve a concentration of 0.58 µmol/mL. This stock solution was further diluted to the concentrations of 12 µg/mL to 8 µg/mL PSMA-1007. It was essential that all dilutions were carried out using “protein low bind” tips as well as tubes. For the determination of the specificity of the method, standard solutions of PSMA-1007 labeling precursor and hydroxy-PSMA-1007 in a concentration of 12 µg/mL were prepared accordingly.

### 3.3. Validation of the HPLC Method—Chemical Purity

#### 3.3.1. Identity and Specificity

For the validation of the chemical identity, the reference standard PSMA-1007 was injected and its retention time determined. The retention times of known impurities like PSMA-1007 labeling precursor and hydroxy-PSMA-1007 were also determined by single injection. The specificity of the method was validated by analyzing a mixture of the reference standard PSMA-1007 and known impurities. Resolution between the peaks was calculated by the Chromeleon 7.2 software and had to be >2.0. Specificity of the method regarding unknown impurities was determined by analyzing the resolution of neighboring peaks to PSMA-1007 in the UV chromatogram of a typical batch of [^18^F]PSMA-1007.

#### 3.3.2. Precision/Repeatability

For the validation of the precision/repeatability of the method, standard solutions of PSMA-1007 at five different concentrations were injected four (UltiMate 3000) or five (ICS5000) times. For each concentration the average of the integrated peak areas as well as its standard deviation (SD) were calculated using Microsoft Excel 2013. From these values the relative standard deviation was determined using the formula: RSD% = (SD/average) × 100. The RSD has to be < 2%.

#### 3.3.3. Linearity

For validation of the linearity of the method, reference solutions of PSMA-1007 over the range of 8–12 µg/mL (80–120% of limit value) were each injected four (UltiMate 3000) or five (ICS5000) times. Linear regression of the average integrated peak areas versus the known concentration was performed using GraphPad Prism 9 software and the correlation coefficient as well as the linear curve equation were determined. The points should lie on a straight line and the correlation coefficient should be ≥ 0.99.

#### 3.3.4. Limit of Quantitation—LOQ

For validation that the method is suitable to determine 80 % of the limit value of PSMA-1007, the signal-to-noise (S/N) ratio in a chromatogram of the standard solution with a concentration of 8 µg/mL was evaluated using Chromeleon 7.2 software. Then the standard solution was diluted further (3 µg/mL, 1.5 µg/mL, 0.75 µg/mL, and 0.3 µg/mL) and analyzed until the S/N ratio was < 10. The last concentration with a S/N > 10 was defined as the LOQ of the method.

#### 3.3.5. Accuracy

In order to validate the accuracy of the method, for each measurement of the samples for the determination of linearity the average bias% was calculated. The average bias% measures the degree of conformity of a value obtained from the analytical method and the accepted true value. For each measurement of a given sample the concentration was calculated using the integrated peak area and the linear equation. This value was compared to the accepted true value of the concentration level (e.g., 8 µg/mL). For the determination of the average bias % of a given concentration level, the deviations of each sample in the level were summed up, divided by the accepted true value and multiplied with 100.

### 3.4. Validation of the HPLC Method—Radiochemical Purity

#### 3.4.1. Radiochemical Identity and Specificity

For validation of the radiochemical identity of [^18^F]PSMA-1007, a sample of a batch of [^18^F]PSMA-1007 injection solution was spiked with non-radioactive reference standard PSMA-1007 and analyzed. The retention time of PSMA-1007 in the UV chromatogram was compared with that of [^18^F]PSMA-1007 in the radiochromatogram. Both peaks have to co-elute with a deviation of < 10% between both retention times. Depending on the arrangement of the UV and radiodetector in the HPLC system the retention time of the radioactive compound is either shifted to higher (1. UV and 2. radio) or lower (1. radio and 2. UV) retention times.

As for validation of the chemical purity, the method also has to be specific for the determination of the radiochemical purity. For this purpose the resolution of the [^18^F]PSMA-1007 peak to known as well as unknown radioactive impurities has to be validated. A known impurity of [^18^F]PSMA-1007 injection solution is unreacted [^18^F]fluoride. In order to analyze the resolution between the peak of [^18^F]fluoride and [^18^F]PSMA-1007, a sample of [^18^F]PSMA-1007 was spiked with [^18^F]fluoride solution under the addition of citrate buffer for reducing tailing of the [^18^F]fluoride peak. The resolution between the resulting peaks in the radiochromatogram was calculated using the Chromeleon 7.2 software and has to be > 1.5. For validation of the specificity to unknown impurities, the resolution of [^18^F]PSMA-1007 to neighboring peaks in a given batch was also calculated using the software.

#### 3.4.2. Recovery

A common problem while determining the radiochemical purity of ^18^F-radiopharmaceuticals may be that using the given analytical method unreacted [^18^F]fluoride may stick to the HPLC column falsifying the results of the analysis. To rule that out, the recovery of a sample of the radiopharmaceutical has to be determined. For this purpose a known radioactive amount of [^18^F]PSMA-1007 injection solution was injected, the whole run was collected and then its radioactive amount was determined using an ionization chamber. After half-life correction of the obtained value both values can be compared and the percentage of recovery can be calculated. The recovery should be > 95%.

#### 3.4.3. Linearity

Linearity of the radiodetectors was validated using two different methods. For the first method an equal volume from the same radiopharmaceutical solution was injected four times at specified time intervals. The time intervals depend on the half-life of the radionuclide so that an expected amount of radioactivity remains in the sample and is injected (e.g., 70%, 50% and 33% of radioactivity). For fluorine-18 the respective time intervals were: t_0_, t_1_ = 56 min, t_2_ = 110 min, and t_3_ = 178 min. The percentage of the integrated peak area was calculated (=(area(t_x_)/area(t_0_)) × 100) and compared to the expected value. From these values the linearity of the detector can be determined by plotting the measured percentage values against the expected percentage values. For the determination of the linearity of the radiodetector of each HPLC system three independent measurement series were evaluated and the correlation coefficients of the linear regressions of the average values were determined.

Another method for determination of the linearity of a radiodetector is the repeated measurement of an equal volume of the same radiopharmaceutical solution at fixed timepoints as realized for the determination of the limit of quantitation. The obtained integrated peak areas are plotted against the expected radioactive concentration of the sample at the timepoint of injection (calculated by half-life correction of the known initial amount) and the correlation coefficient of the linear regression can be determined.

#### 3.4.4. Limit of Quantitation

The determination of the limit of quantitation of the radiodetectors was realized by consecutive measurements of the same radiopharmaceutical solution. Due to the radioactive decay the measured integrated peak areas decrease over time resulting in smaller S/N ratios with every measurement. The S/N ratio was determined using Chromeleon 7.2 software and the limit of quantitation was the last radioactive concentration which fulfilled the criterion of a S/N > 10. The radioactive concentration at a given timepoint was calculated using the decay equation.

#### 3.4.5. Intermediate Precision

For the determination of the intermediate precision of the method concerning re-integration of the radiochromatograms, 10 batches of [^18^F]PSMA-1007 injection solution were integrated independently by 3 different analysts. For all batches the relative standard deviation of the results regarding the radiochemical purity of [^18^F]PSMA-1007 by the different operators was calculated using Microsoft Excel 2013. 

## 4. Conclusions

For the use of a radiotracer in human applications its production as well as its quality control have to be GMP-compliant. In this study we developed, optimized and validated a fast and reliable method for the routine determination of the chemical and radiochemical purity of [^18^F]PSMA-1007, a tracer for imaging of prostate cancer, by radio-HPLC. The described radio-HPLC method fulfilled all acceptance criteria—for e.g., specificity, linearity, and accuracy—and is therefore well suited for the routine quality control of [^18^F]PSMA-1007 before release of the radiopharmaceutical. Albeit it is different to the method recently published in a European Pharmacopeia monograph, it has certain advantages, not least of all an easier technical implementation. Therefore, this method is an attractive alternative for users all over the world. Even though European users have to consider the monograph for [^18^F]PSMA-1007, they may use this method when explaining its advantages to their authorities and showing that it is suitable. The here described method was successfully validated on several radio-HPLC systems in our lab and used for the analysis of more than 60 batches of [^18^F]PSMA-1007. Preparation of the PSMA-1007 standard solutions in PBS proved essential for the ICS5000 system and beneficial for the UltiMate 3000 system. Using this method, the chemical and radiochemical purity of [^18^F]PSMA-1007 can routinely be evaluated assuring patient safety.

## Figures and Tables

**Table 1:** Recommended acceptance criteria for the chemical and radiochemical purity of [^18^F]PSMA-1007 injection solution determined by radio-HPLC.

Parameter	Acceptance Criteria
Chemical Purity	PSMA-1007: ≤0.1 mg/V_max_^#^ ≙ 10 µg/mL
	Any other impurity: ≤ area of reference peak *
	Sum of all impurities: ≤ 5 × area of reference peak *
	Disregard limit for peak areas ≤ 0.3 × area of reference peak *
Radiochemical Purity	≥95%

^#^ Vmax was determined as 10 mL per patient, * peak area of reference solution PSMA-1007 (10 µg/mL) has to be determined for comparison.

**Table 2:** Parameters of the radio-HPLC method.

**Column**	Precolumn: Chromolith Performance RP-18e (10 × 4.6 mm) (Merck; Germany) Column: Chromolith Perfomance RP-18e (100 × 4.6 mm) (Merck; Germany)
**Column temperature**	Room temperature
**Injection volume**	25 µL
**Detector 1**	UV detector @ 254 nm
**Detector 2**	Radioactivity detector
**Mobile Phase**	A: Acetonitrile, B: 0.1% TFA in water
**Gradient**	Start 5% A; till 1.5 min increasing to 15% A; till 10.5 min to 35 % A; till 13 min to 95% A; till 19 min back to 5% A
**Flow rate**	3 mL/min
**Run time**	19 min

**Table 3:** Parameters of the radio-HPLC method suggested in monograph 3116 [17].

**Column**	Solid core octadecylsilyl silica gel for chromatography (2.7 µ)
**Column temperature**	30 °C
**Injection volume**	20 µL
**Detector 1**	UV detector @ 225 nm
**Detector 2**	Radioactivity detector
**Mobile Phase**	A: Acetonitrile, B: sodium dihydrogen phosphate (3.12 g/L) in water (pH 2.5 with phosphoric acid)
**Gradient**	Start 23% A; 2–14 min increasing to 30% A; 14–17 min increasing to 60% A; till 21 min remaining at 60% A
**Flow rate**	1.3 mL/min
**Run time**	>21 min (≈25 min)

**Table 4:** Parameters and acceptance criteria for the validation of the radio-HPLC method. Abbreviations: RSD: relative standard deviation, R^2^: correlation coefficient, S/N: signal-noise-ratio.

Chemical Purity (UV detector)	
**Parameter**	**Acceptance criteria**
Specificity	Resolution > 2.0
Precision/Repeatability	RSD < 2%
Linearity	*R*^2^ ≥ 0.99
LOQ	S/N > 10
Accuracy	Average bias < 5%
**Radiochemical Purity (radiodetector)**	
**Parameter**	**Acceptance criteria**
Identity	Difference t_R_ ≤ 5%
Specificity	Resolution > 1.5
Recovery	> 95%
Linearity	*R*^2^ ≥ 0.99
LOQ	S/N > 10
Intermediate Precision	RSD < 2%

**Table 5:** Integrated peak areas for different concentrations of PSMA-1007 in physiological saline solution.

UltiMate 3000		
Concentration PSMA-1007 µg/mL	Integrated Peak Areas Average ± SD mAU*min	RSD%
**8**	0.9313 ± 0.0024	0.26
**9**	1.0632 ± 0.0010	0.10
**10**	1.1667 ± 0.0024	0.21
**11**	1.2842 ± 0.0003	0.03
**12**	1.3992 ± 0.0025	0.18
**ICS5000**		
**Concentration** **PSMA-1007** **µg/mL**	**Integrated Peak Areas** **Average ± SD** mAU*min	**RSD%**
**8**	0.3781 ± 0.0006	0.17
**9**	0.4493 ± 0.0031	0.70
**10**	0.4851 ± 0.0019	0.38
**11**	0.5445 ± 0.0018	0.34
**12**	0.5965 ± 0.0020	0.34

**Table 6:** Integrated peak areas for different concentrations of PSMA-1007 in PBS solution.

UltiMate 3000		
Concentration PSMA-1007 µg/mL	Integrated Peak Areas Average ± SD mAU*min	RSD%
**8**	0.9085 ± 0.0025	0.27
**9**	1.0229 ± 0.0021	0.20
**10**	1.1400 ± 0.0019	0.17
**11**	1.2495 ± 0.0011	0.09
**12**	1.3703 ± 0.0058	0.42
**ICS5000**		
**Concentration** **PSMA-1007** **µg/mL**	**Integrated Peak Areas** **Average ± SD** mAU*min	**RSD%**
**8**	0.7677 ± 0.0062	0.80
**9**	0.8748 ± 0.0050	0.57
**10**	0.9807 ± 0.0108	1.10
**11**	1.0917 ± 0.0047	0.43
**12**	1.1932 ± 0.0066	0.55

**Table 7:** S/N ratios for determination of LOQ for PSMA-1007.

Concentration PSMA-1007 µg/mL	UltiMate 3000		ICS5000	
	NaCl	PBS	NaCl	PBS
8	239	565	64	248
3	91	91	38	143
1.5	47	39	17	53
0.75	23	18	9.5	22
0.3	7.2	6.9	-	9.6

**Table 8:** Calculated concentrations and average bias for different concentrations of PSMA-1007 in physiological saline solution.

UltiMate 3000		
Concentration PSMA-1007 µg/mL	Calculated Amount Average ± SD µg/mL	Average bias%
**8**	7.94 ± 0.02	−0.71
**9**	9.09 ± 0.01	0.97
**10**	9.98 ± 0.02	−0.19
**11**	10.99 ± 0.001	−0.05
**12**	11.99 ± 0.02	−0.06
**ICS5000**		
**Concentration** **PSMA-1007** **µg/mL**	**Calculated Amount** **Average ± SD** **µg/mL**	**Average bias%**
**8**	7.90 ± 0.01	−1.26
**9**	9.23 ± 0.07	2.53
**10**	9.90 ± 0.02	−1.04
**11**	11.00 ± 0.03	0.03
**12**	11.97 ± 0.02	−0.22

**Table 9:** Calculated concentrations and average bias for different concentrations of PSMA-1007 in PBS solution.

UltiMate 3000		
Concentration PSMA-1007 µg/mL	Calculated Amount Average ± SD µg/mL	Average Bias%
**8**	8.00 ± 0.02	0.04
**9**	9.00 ± 0.02	−0.03
**10**	10.02 ± 0.02	0.15
**11**	10.97 ± 0.01	−0.27
**12**	11.98 ± 0.05	−0.15
**ICS5000**		
**Concentration** **PSMA-1007** **µg/mL**	**Calculated Amount** **Average ± SD** **µg/mL**	**Average Bias%**
**8**	8.00 ± 0.06	−0.03
**9**	9.00 ± 0.06	−0.01
**10**	9.99 ± 0.10	−0.09
**11**	11.03 ± 0.04	0.28
**12**	11.98 ± 0.06	−0.16

**Table 10:** Comparison of batch results for [^18^F]PSMA-1007 for both HPLC systems.

Batch	Amount PSMA-1007/µg/mL		RCP/%	
	UltiMate 3000	ICS5000	UltiMate 3000	ICS5000
180129-1	6.42	6.28	99.23	99.25
180219-1	7.99	8.13	99.00	98.99
180305-1	11.05	10.56	99.06	99.03
180312-1	10.03	10.01	99.19	99.09
180314-1	9.14	9.10	99.12	99.22

## Data Availability

The data presented in this study are available on request from the corresponding author.
